# Supplementary material for: Complexome profiling of the Chlamydomonas psb28 mutant reveals TEF5 as an early PSII assembly factor
Source: Plant Cell. 2025 Mar 17;37(6):koaf055. doi: 10.1093/plcell/koaf055 (PMC12164590; doi:10.1093/plcell/koaf055)
Supplement: koaf055_Supplementary_Data [file koaf055_supplementary_data.zip › psb28_interactive.zip!/plots/intensity/qualityPlots/peptides/SCLA1_Cre03.g193850.t1.2.html]

### Cre03.g193850.t1.2

Trivial name: SCLA1  
SeqCoverage [median]: 34.30  
Mass: 34.32 kDa  
  
 

| Sequence | MissedCleavage | Mass [Da] | wtIntensity | muIntensity | Score |
| --- | --- | --- | --- | --- | --- |
| ALEEAGVTVTR | 0 | 1144.61 | 18273.97 | 47904.34 | 150.08 |
| GGTSHLGLPVFNTVAEAK | 0 | 1796.94 | 394.44 | 0.00 | 44.01 |
| GTATDKIK | 1 | 832.47 | 576.05 | 0.00 | 119.41 |
| IGIMPGYIHTPGK | 0 | 1382.74 | 0.00 | 4890.07 | 79.82 |
| KGGTSHLGLPVFNTVAEAK | 1 | 1925.04 | 1008.00 | 8580.61 | 59.28 |
| KPVVSFIAGLTAPPGR | 1 | 1608.94 | 3527.09 | 3680.67 | 58.67 |
| MGHAGAIISGGK | 0 | 1097.57 | 712.39 | 7709.92 | 60.49 |
| NGTFHTEQAIAYGTQMVGGVTPK | 0 | 2406.16 | 0.00 | 1526.61 | 91.24 |
| SPAQMGVTMMR | 0 | 1207.55 | 2774.38 | 5385.60 | 124.10 |
|  |  |  | 27266.3 | 79677.8 |  |
